# The Amsterdam Studies of Acute Psychiatry I (ASAP-I); A prospective cohort study of determinants and outcome of coercive versus voluntary treatment interventions in a metropolitan area

**DOI:** 10.1186/1471-244X-8-35

**Published:** 2008-05-14

**Authors:** Louk van der Post, Robert Schoevers, Vincent Koppelmans, Irene Visch, Clemens Bernardt, Niels Mulder, Aartjan Beekman, Lieuwe de Haan, Jack Dekker

**Affiliations:** 1 JellinekMentrum Mental Health Care, Amsterdam, The Netherlands; 2 VU University Medical Center, Department of Psychiatry, Amsterdam, The Netherlands; 3 VU University, Faculty of Psychology and Pedagogy, Department of Clinical Psychology, Amsterdam, The Netherlands; 4 Erasmus MC, University Medical Center, Rotterdam, Department of Psychiatry, The Netherlands; 5 University of Amsterdam Medical Centre, Department of Psychiatry, The Netherlands

## Abstract

**Background:**

The overall number of involuntary admissions is increasing in many European countries. Patients with severe mental illnesses more often progress to stages in which acute, coercive treatment is warranted. The number of studies that have examined this development and possible consequences in terms of optimizing health care delivery in emergency psychiatry is small and have a number of methodological shortcomings. The current study seeks to examine factors associated with compulsory admissions in the Amsterdam region, taking into account a comprehensive model with four groups of predictors: patient vulnerability, social support, responsiveness of the health care system and treatment adherence.

**Methods/Design:**

This paper describes the design of the Amsterdam Study of Acute Psychiatry-I (ASAP-I). The study is a prospective cohort study, with one and two-year follow-up, comparing patients with and without forced admission by means of a selected nested case-control design. An estimated total number of 4,600 patients, aged 18 years and over, consecutively coming into contact with the Psychiatric Emergency Service Amsterdam (PESA) are included in the study. From this cohort, a randomly selected group of 125 involuntary admitted subjects and 125 subjects receiving non-coercive treatment are selected for further evaluation and comparison.

First, socio-demographic, psychopathological and network characteristics, and prior use of health services will be described for all patients who come into contact with PESA. Second, the in-depth study of compulsory versus voluntary patients will examine which patient characteristics are associated with acute compulsory admission, also taking into account social network and healthcare variables. The third focus of the study is on the associations between patient vulnerability, social support, healthcare characteristics and treatment adherence in a two-year follow-up for patients with or without involuntarily admittance at the index consultation.

**Discussion:**

The current study seeks to establish a picture of the determinants of acute compulsory admissions in the Netherlands and tries to gain a better understanding of the association with the course of illness and patient's perception of services and treatment adherence. The final aim is to find specific patient and health care factors that can be influenced by adjusting treatment programs in order to reduce the number of involuntary admissions.

## Background

The overall number of involuntary admissions has increased in a number of European countries, in particular in Germany, France, England, Austria, Sweden and Finland [1-3]. In the Netherlands, the number of compulsory admissions has doubled between 1979 and 2004, rising from 23 to over 53 per 100.000 inhabitants [4]. This increase includes both compulsory admissions in crisis situations without reference to the courts ("compulsory admissions") and compulsory admissions after recourse to the courts ("court orders") [4]. In the Amsterdam area, the number of compulsory admissions even rose by 319% to 86 per 100,000 in the period between 1979 and 2004 [5]. As a result, the proportion of involuntary admitted patients to Psychiatric Intensive Care Units is now around 80% [6]. These developments imply that patients with severe mental illnesses more often progress to stages in which acute, coercive treatment is warranted. It is clear that this is an undesirable trend, which not only leads to a very negative experience for the patient and a reduction of his autonomy but also has a negative effect on the prognosis of these disorders. When intervening at a later stage of decompensation, psychotic episodes take longer to remit, and the restoration of premorbid functioning is less optimal in comparison with early intervention [7,8].

**Figure 1:**
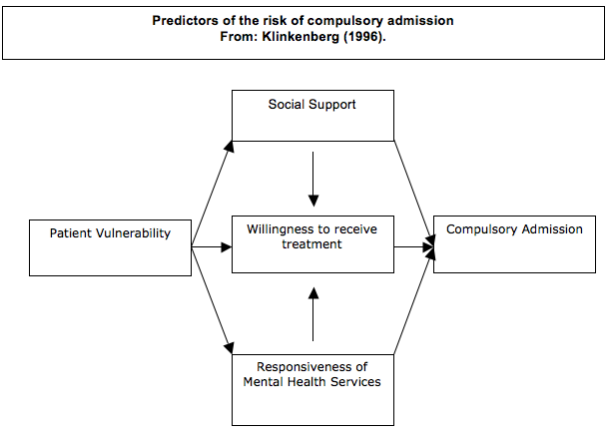
Predictors of the risk of compulsory admission.

According to Klinkenberg a number of interacting factors are believed to be associated with the risk for coerced admission (See Figure 1) [9]. *Patient vulnerability *can be defined by individual patient characteristics such as type and severity of psychopathology, and socio-demographic factors. Complex mental disorders, defined as combinations of psychotic, affective or personality disorders with addiction, and/or behavioral problems are more often found in urbanized areas and constitute a challenge for both psychiatric, general medical, social and community facilities of larger cities [10,11]. Furthermore, specific cultural and socio-economic groups as for example migrants show morbidity rates that may be substantially elevated in comparison with others [12]. These patients appear to be underserved by the mental health care system, and more often have their first contact with mental health workers through the emergency services [13,14].

A second factor determining risk of coercive admission is *social support*. Higher levels of social support may reduce the risk of (compulsory) admission. The availability of social support can be determined by variables such as 'living alone/together', having family contact, but may also be defined by a proxy measure such as income level or the availability of resources. Differences in compulsory admission rates between regions in the UK could in part be explained by differences in socio-economic-deprivation of the population measured with the Mental Illness Needs Index [15]. However, very few studies have examined the extent to which direct (social) support of the patient from family or partner may play a specific role in determining the risk of emergency admission.

As a third factor, health care characteristics, or the *'responsiveness' of the health care system *[9], may be related to the increase in coercive measures. Over the last 20 years, mental health care in western countries has changed from a hospital based to a community based system, with significant reductions of clinical facilities and the development of various types of community mental health teams [16]. It has been argued that this 'deinstitutionalisation', with a reduced length of in-patient treatment, has taken place at the expense of more frequent readmissions [1,17]. In addition, a decreasing societal tolerance for deviant behaviour and a growing opinion that respecting patient's rights and autonomy may not be an excuse for neglect of those in need, may play a role in the development of more coercive treatment strategies [18,19].

A fourth factor determining the risk for acute admission is patient's *treatment adherence*. In psychiatry, as in the whole of medicine, treatment non-adherence is a major problem with very significant implications for the delivery of adequate care, patient prognosis and health care costs [20-22]. Studies examining treatment adherence of patients using antipsychotic medication show non adherence rates of 40% to 50% [23]. It is estimated that, in patients with schizophrenia, maintenance therapy reduces the risk of relapse by about two-thirds [24,25]. A meta-analysis of data from several large collaborative studies showed that the number of people who survive without relapse after discontinuing drug treatment declines exponentially by around 10% a month [26,27].

It thus appears that, for various reasons, a substantial number of patients may not be treated in an adequate and timely fashion, which may have significant implications for both the quality of care and the personal and societal costs associated with mental health problems. As a result, the demand on psychiatric emergency services, that in many areas function as the emergency gate keeper of the mental health system, is increasing. Still, the number of studies that have examined these developments, and the possible consequences in terms of optimizing health care delivery in emergency psychiatry, is strikingly small. Furthermore, existing studies have important methodological limitations [28].

Drawing on the model designed by Klinkenberg (See Figure 1), the current study seeks to examine the factors associated with compulsory admission in the Amsterdam region, and the relationship between compulsory admission, patient prognosis and future treatment adherence over a two-year period [9]. The study includes both a large cohort consisting of all consecutive patients who come into contact with the Psychiatric Emergency Service Amsterdam (PESA) with two-year follow-up, and an in-depth assessment of a smaller but representative sample consisting of patients who are coercively admitted and patients who are not coercively admitted.

## Methods

### Aims

The study has three main goals:

1) To determine socio-demographic, psychopathological and network characteristics as well as prior use of mental health care of a large cohort of patients who come into contact with emergency psychiatric services.

2) To determine which of these characteristics are associated with acute compulsory admission and to examine whether social network and health care variables modify this association.

3) To examine the associations between patient vulnerability, social support, health care characteristics and treatment adherence in a two-year follow-up, with a special emphasis on the comparison of patients who were and those who were not involuntarily admitted at the index consultation.

### Study design

The study is a prospective cohort study consisting of all patients who consecutively come into contact with PESA (Wave 1), with a more detailed assessment of a randomly selected nested cohort of patients who are involuntarily admitted as a result of the index consultation and a control group of patients who are not involuntarily admitted. The study has a one and two-year follow-up.

The baseline cohort for Wave 1 is composed of all consecutive patients receiving emergency consultations by PESA in the period from 15 September 2004 to 15 September 2006. Every patient who came into contact with these acute services was automatically included in Wave 1. The baseline cohort thus consists of around 4600 consultations in total, of which roughly 600 were expected to result in compulsory admission.

Subsequently, for Wave 2, two groups of 125 subjects were randomly selected from the patients from Wave 1, one group of coercively admitted patients and one group in whom consultation did not result in compulsory admission. In the group of voluntary (treated) patients, consultation resulted in either (voluntary) admission, outpatient treatment, admission to a crisis centre, or no further intervention. For Wave 2, patients who are not residing in the Amsterdam catchment area (in total approximately 15%) are excluded for pragmatic reasons.

**Figure 2:**
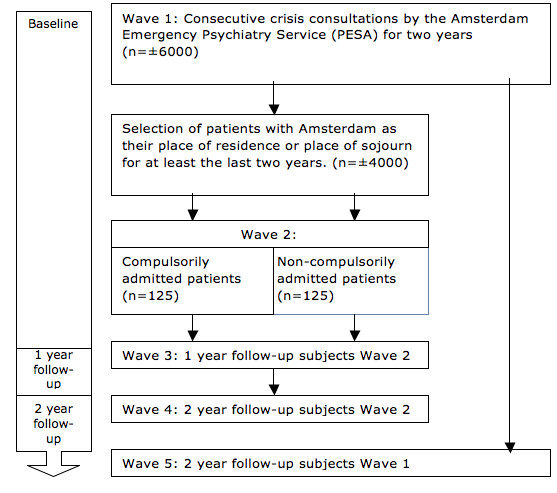
Flowchart.

Waves 3 and 4 are the one and two-year follow-up measurements for these 250 subjects, and Wave 5 is a two-year follow-up of the full cohort (See Figure 2: flow chart).

### Participants

Emergency Public Mental Health Care in Amsterdam is delivered by both the City Mental Health Service Amsterdam (CMH) and by PESA. At first, a socio-psychiatric nurse from the CMH performs a psychiatric screening of persons who come into contact with the police or other general (community) services [29]. Patients in need of immediate psychiatric consultation are then referred to PESA. PESA provides 7 × 24 city-wide acute psychiatric services and combines a well staffed and secure facility for acute psychiatric assessment with outreach activities such as home visits. PESA has the sole mandate for emergency admission to all of the Amsterdam Psychiatric Intensive Care Units (PICU) outside office hours. In addition, five local community mental health teams cater for emergency services during office hours in their district. These teams mostly work in response to requests from GPs and mental health staff in their catchment area.

All patients are examined by a psychiatrist or a resident in psychiatry and a community mental health nurse to determine the need and urgency for psychiatric treatment. Compulsory admission is one of the possible outcomes of this index consultation. If the patient already engages in treatment, his or her own mental health worker or GP will be contacted for referral or consultation.

All patients aged 18 years and above who come into contact with PESA are included in the baseline assessment of this study. For wave 2, a total of 250 patients are randomly selected from the wave 1 cohort. To obtain a representative sample, every patient with involuntary admittance is approached, and of those for whom the index consultation did not result in forced admission, every 8^th ^patient is approached.

### Informed consent and data security

As the data for Wave 1 are routinely gathered in clinical practice, informed consent is not needed. Additional data on earlier history and service use are gathered through anonymous pairing by means of existing medical registration systems. Subjects randomly selected for Wave 2 receive a letter with the request to participate in further assessments (Additional files 1 &2). In follow-up to this request, patients are contacted by a research employee either by telephone, in writing or by actually visiting the patient at his or her place of residence. Patients then receive information verbally as well as in writing, before they are asked to sign an informed consent. The whole procedure was endorsed by the Medical Ethical Committee for Mental Health Care Institutions (METIGG) (Additional file 3).

Confidential information and participant names are secured by the medical confidentiality rules and are treated according to the code of conduct for medical research, developed by the FMWV (the Federation of Biomedical Scientific Societies).

The results of the participant questionnaires are not accessible to Mental Health workers. All study related documents and data are stored on a protected central server from the research department of JellinekMentrum Mental Health Care Amsterdam. Only members of the research group have access to the respective files.

### Measurements

Table 1 provides an overview of the assessments at the different waves of the study.

**Table 1:** Outcome measures of the ASAP study

**Measure**	**Wave**				
	**1**^t0^	**2**^t0^	**3**^t1^	**4**^t2^	**5**^t2^

***Questionnaire***					

ASR; section social support		X	X	X	
BES		X	X	X	
FFPI ; impulsiveness, vulnerability and anger/hostility scales		X		X	
LTE-Q		X	X	X	
Morisky		X	X	X	
MHP-P; section friends and family relations		X	X	X	
NEO-PI-R		X		X	
NPV		X			
SAI		X	X	X	
SES		X	X	X	
SPI	X				X
UCL		X			
VSSN		X	X	X	
VSSS; European version		X	X	X	
VSSS-family		X	X	X	

***Variable***					

Contact between patient and mental health care system		X	X	X	
Contact between support system and mental health care system		X	X	X	
Date of first mental health care subscription	X	X			
Demographic information (Date of birth, gender, ethnicity)	X	X	X	X	
Domestic situation	X	X	X	X	X
DSM IV criteria including GAF score	X				X
Extent of mastering the Dutch language	X				
Information about preceding compulsory admissions		X	X	X	
Intervention at completion of the consultation	X				
Mental health care services used in the preceding year			X		X
Number of friends/family present at crisis consultation	X				
Number, frequency and place of treatment sessions during the three months preceding the consultation	X				
Size of current social support system	X				X
Socio-Economic Status		X	X	X	

#### Wave 1

##### Patient characteristics

demographic variables (age, gender, domestic situation, ethnicity), clinical psychiatric diagnosis categorized according to DSM-IV, and severity of the actual psychopathology according to the Severity of Psychiatric Illness rating scale (SPI) [30].

##### Social support

is assessed by noting the number of friends and family who are present at the crisis consultation or who are involved through telephone contact during consultation.

##### Previous health care use

date of first mental health care subscription, and information on number, frequency and type of treatment during five years preceding crisis consultation. This is subdivided into five categories: overall mental health care during the preceding five years, and more detailed information on mental health care use in the periods between 2 and 3 months preceding consultation; between 1 and 2 months preceding consultation; between 1 and 4 weeks, and during the last week before the index consultation. 

##### Intervention

the intervention resulting from consultation is obtained from the Electronic Patient File (EPD) and the hospital registration database.

#### Wave 2

Wave 2 constitutes a more detailed measurement of both patient and health care characteristics. These are determined in interviews conducted by a trained researcher with the patient, with a member of their social network and with the treating mental health professional, if available.

##### Patient Characteristics

occupational status, time residing at abode, information regarding previous (compulsory) admissions. Concerning major life events preceding crisis consultation are measured with the List of Threatening Experiences (LTE-Q) [31]. Personality is assessed using the Five Factor Personality Inventory (FFPI) [32], the NEO Personality Inventory (NEO-PI-R) [33] and the Dutch Personality Questionnaire (NPV) [34]. Coping style is measured by means of the Utrecht Coping List (UCL) [35]. As the overall number of questions was high, both the burden on patients and the overall costs dictated limiting the number of items from some of the instruments, thus including only the core domains of interest for this study.

##### Social support

The social support system is assessed using the Social Network Structure Questionnaire (VSSN) [36]. The frequency of contacts, and the perception of social support as perceived by the patient, is measured with the Multidimensional Health Profile – Psychosocial Functioning (MHP-P); Friends and Family Relationships (Dutch translation) [37] and the Adult Self Report (ASR) 'social support' scale (Dutch translation) [38,39]. The opinion of the patients' partner or relatives is assessed by 15 questions of the Involved Evaluation Scale (BES) [40] and the family version of the Verona Service Satisfaction Scale (VSSS; European version and Family version) [41]; (Dutch translation Family Version according to authorized translation patient version: L. van der Post, 2004).

##### Previous health care use

The Verona Service Satisfaction Scale (VSSS) is used to explore the opinion of both patient and family or partner regarding the quality of treatment preceding the index consultation.

##### Intervention

In addition, the VSSS will be used: a) to define which health care professionals the patient came into contact with, and if there have been changes in personnel involved with the patient; b) to determine the amount and frequency of contacts between health care professionals and the current social support- system; c) to record the extent of agreement and cooperation between patient and health care professionals in relation to type and objective of treatment; and d) to assess the relationship between treatment, the quality of social support and the willingness to accept treatment and cooperate with health care professionals. The extent to which there was agreement between patient and health care professionals regarding the aim and type of treatment was assessed by means of the Schedule for Assessment of Insight (SAI-NL) [42]. Opinions about and subjective experiences with medication was determined by the Morisky [43] and the Drug Attitude Inventory (DAI-10) [44].

### Instruments

#### SPI [30]

The Severity of Psychiatric Illness rating scale is a decision support tool for psychiatric hospital admissions which was developed and validated to provide reliable, clinically relevant information to providers and case managers. The SPI provides a structured description of the severity of psychopathology and possible complications regarding the disorder and regarding treatment. These three fields contain in a total of 14 items which are rated on a 4-point scale from 0 tot 3, with 0 indicating no problems and 3 indicating an extreme problem. Completing the instrument takes five to ten minutes. Validity proved to be good.

#### FFPI [45]

The Five Factor Personality Inventory is a self-report instrument assessing the Big Five factors of personality [46]. It consists of 100 brief and concrete statements that subjects can agree or disagree with, with scores on a 5 point Likert-scale). It can be administered in 10–15 minutes. In addition to the five factor scores, the FFPI may be used to assess 40 bipolar facet scores that arise as blends of the Big Five, for the purpose of communicating more specific information about an individual's position in the five-space (applied setting). The five FFPI scales are extraversion, agreeableness, conscientiousness, emotional stability and autonomy. The FFPI received judgments of 'good' and 'sufficient' from the Dutch Documentation of Test and Test research (COTAN) concerning validity and reliability respectively.

#### NEO-PI-R [47]

The NEO-PI-R is a self-report instrument assessing narrow, specific personality traits combining to define broad, global personality factors [46]. It consists of 240 brief and concrete statements that subjects can agree or disagree with, with scores on a 5 point Likert-scale). It can be administered in 40–50 minutes. The Revised NEO Personality Inventory assesses personality at both levels, with six specific facet scales in each of five broad domains, designed to operationalize the five-factor model of personality (FFM) [48,49] neuroticism, extraversion, openness, agreeableness and conscientiousness. The NEO-PI-R received judgments of 'good' and 'sufficient' from the COTAN concerning validity and reliability respectively.

#### UCL [35]

The Utrecht Coping List is a Dutch self-inventory questionnaire with 47 items on a 4-point Likert scale. The UCL measures the way in which people react to situations in which adaptation is required in cognitive, behavioral as well as emotional terms, defining coping as a personality trait. There are 7 subscales: 1) active problem solving 2) palliative reacting 3) evading 4) searching for social support 5) passive reacting 6) expression of emotions and 7) comforting thoughts. Administration takes about 10 minutes. Both validity and reliability have been judged sufficient by the COTAN.

#### LTE-Q [31]

The List of Threatening Experiences is a 12-event self-inventory initially modified by Bebbington and colleagues from a 67 life-events inventory introduced by Tennant and Andrews [50]. Each event is covered by 1 item with scores on a 2 point Likert scale (yes or no). Subsequently the number of months before the event took place can be administered. The time to complete the list takes approximately 10 minutes. The categories inquire about recent adverse experiences in personal relationships, employment, illness, and financial and legal issues in the last six months. Reliability and validity proved to be good [51].

#### NPV [34]

The Dutch Personality Questionnaire (NPV) is a self inventory list assessing personality aspects. The NPV consists of 132 item plus one instruction item with scores on a 3 point Likert scale; yes, no or unknown. The items are divided into seven main categories, based on personality features: inadequacy, social inadequacy, rigidity, discontentedness, complacency, dominance and self-esteem. Factor analysis uncovered three underlying dimensions: neuroticism, extraversion and dogmatism. Administration approximately takes 20–30 minutes. According to the COTAN, reliability and validity proved to be sufficient.

#### VSSN [36]

The Social Network Structure Questionnaire is a Dutch inventory list with ten questions about the persons forming the patients natural network: which persons (family, neighbors, work) are playing a role in the patients life and how often. It also has a picture with inner and outer circles in which the patient has to depict the position of these persons in relationship to him/herself. The list can be administered in 15–20 minutes. Information about the psychometric characteristics of this questionnaire are not available.

#### MHP-P [37]

The Multidimensional Health Profile – Psychosocial Functioning is a self-report screening instrument for use in mental health and primary care settings. The MHP was designed to alert health care personnel to potential problem areas that should be addressed in more detail. The MHP-P (58 items) covers four basic areas: Life Stress, Coping Skills, Social Resources, and Mental Health. The MHP utilizes different test formats, although 5-point rating scales are used wherever possible. Administering the MHP-P takes about 15 minutes. Research has revealed preliminary evidence of convergent and discriminant validity.

#### ASR [38,39]

The Adult Self Report form is a self report questionnaire for adults from 18 to 59 years old exploring several aspects of mental health. The ASR starts with questions regarding background and continues with 126 items, of which 123 are scored on a 3 point Likert scale from 0 to 2 (not true, somewhat true, very true) and 3 open answer questions. Administration roughly takes roughly 5–20 minutes. The profiles for scoring the ASR include normed scales for Adaptive Functioning, Empirically Based Syndromes, Substance Use, Internalizing, Externalizing, and Total Problems. In addition, the profiles feature new DSM-oriented scales consisting of items that experts from 10 cultures identified as being very consistent with DSM-IV categories. The ASR consists of the following empirically based scales: Substance Use, Critical Items, Internalizing, Externalizing, and Total Problems. The DSM-oriented scales are: Depressive Problems; Anxiety Problems; Somatic Problems; Avoidant Personality Problems; Attention Deficit/Hyperactivity Problems; and Antisocial Personality Problems.

#### BES [40]

The Dutch Involved Evaluation Scale is a self-report scale derived from the Burden on the Family questionnaire [52] and was developed to assess the effects of severe psychiatric disorders on the family in overall daily living. We only use the first 15 questions from the first section of this scale: patient and respondent characteristics and nature and intensity of their relationship. The list consists of multiple-choice questions and some questions regarding patient characteristics. Mean administration time of this shortened version is 5 to 10 minutes. Research showed the BES to be highly sensitive with satisfactory validity and satisfactory to good reliability.

VSSS-EU (European version and Family version; Dutch translation Family Version according to authorized translation patient version: L. van der Post, 2004) [41] The Verona Service Satisfaction Scale European Version is a method to measure satisfaction with psychiatric services and was developed from the Italian VSSS-54 version, which was translated into Dutch. Specific items were changed to adapt them to the context of the Dutch mental health system. Conceptually, the items in the VSSS-EU cover seven dimensions: Overall Satisfaction, Professionals' Skills and Behavior, Access, Efficacy, Types of Intervention and Relative's Involvement. Each conceptual dimension consists of three items which cover general aspects of satisfaction with services: a) Overall; b) Professionals' skills and behavior; c) Information on services, disorders and therapies; d) Access to location, physical layout and costs; e) Overall-, symptom-, social skills- and family efficacy; f) Care; and g) Patient's satisfaction with help given to his or her closest relative. Questions concern the past year. Items 1–40 are on a 5 point Likert scale, items 41–54 consist of three standard questions. The instrument is designed for self-administration and can be completed in 20–30 minutes. Research from Ruggeri et al. (2000) proved the VSSS-EU to be reliable and valid.

#### SAI-NL [42]

The Schedule for Assessment of Insight-NL is a Dutch translation of the SAI, which is a semi-structured interview that measures three dimensions of insight (treatment compliance, recognition of illness, and relabeling of psychotic phenomena), as well as awareness of changes in mental functioning, of the need for treatment and of the psychosocial consequences of illness. It also includes a question on response to hypothetical contradiction. The SAI is administered to psychotic patients and the administration time takes roughly 10 minutes. The list consists of 9 items, of which the first 6 are scored on a 3 point Likert scale and the last 3 on a 5 point Likert scale. Higher scores indicate a higher level of insight. Research has demonstrated a significant correlation between the SAI and other insight scales.

#### Morisky [43]

This medication adherence scale is a self-report tool for (the complexity of) medication compliance behavior. It is quick and simple since it only contains 4 questions that require a Yes or No answer. Study results suggest the Morisky to be both reliable and valid.

#### SES (Dutch translation: Van Baars & Mulder 2004) [53]

This Service Engagement Scale has 14 items divided into four fields: availability, collaboration, help seeking and treatment adherence. The items are rated 0 (not at all or rarely), 1 (sometimes), 2 (often) or 3 (most of the time). The list is administered by a nurse or other closely involved health care professional and completing the list takes approximately 5–10 minutes. It has been shown that this scale identifies individuals who are experiencing difficulties in engaging with mental health care services. Reliability and validity were successfully tested by Tait et al. [54].

### Data analysis and statistical power

We will first use simple statistics to describe the socio-demographic, psychopathological and network characteristics of, and the prior use of health services by, the large cohort of patients who come into contact with emergency psychiatric services. We will then use logistic regression to determine the variables most closely linked to the outcome measure for the study, viz. voluntary or compulsory treatment. We will calculate the 'best-fitting model' in order to identify a prediction model for use in the clinic with a limited number of variables that provides sufficiently accurate predictions for the outcome measures of the study. These analyses will also determine the relative risks and the attributive risks for the individual variables.

The differences between the group of voluntary patients and compulsory admitted patients (from the in-depth study) in terms of patient vulnerability, social support, health care characteristics and treatment adherence in the two-year follow-up will be investigated using chi-square tests, ANOVA/ANCOVA, and MANOVA/MANCOVA. Missing data will be replaced using sophisticated imputation techniques such as multiple imputation or regression imputation. Logistic regression will be performed to identify the variables most strongly associated with the outcome measures of the study.

We expect a clinically relevant difference between the voluntary and compulsory groups, and an effect size of approximately 0.5. To detect a difference of this kind in effectiveness between the groups with α = .05 and β = .80, 64 respondents are needed in each group. Since we expect a considerable level of drop-out (50%) between the inclusion stage and the final follow-up measurement, we estimate that 125 patients per group will be required initially.

## Discussion

The current study seeks to deepen our understanding of the factors associated with compulsory admissions in the Amsterdam area. By comparing patients with and without compulsory admissions on both patient, social and health care characteristics, and through a detailed follow-up of service use and treatment outcome, we hope to increase our knowledge on factors associated with compulsory admissions and associations with the course of illness, with patient's perception of services and treatment adherence.

### A preliminary reflection on the limitations and strengths of our design

A limitation of this design is that it will be difficult to weigh the specific contributions of the various elements determining compulsory admission and acute service use. In this sense, the study will provide descriptive data. Still, to date very few studies have been able to systematically collect a comprehensive dataset consisting of factors associated with the risk for coercive admission [28,55].

Another limitation may be that the patient group using emergency services is by nature difficult to engage in studies such as these. Although wave 1 uses data gathered in day to day mental health care and is therefore not affected by any form of selection bias, there is a risk of bias in Wave 2, in which informed consent is needed. This can in part be countered by comparing those patients that participate in Waves 2, 3 and 4 with the full cohort in Waves 1 and 5, and to determine possible differences in both patient, social and health care characteristics and course of illness. This will enable a better interpretation of the generalizability of the results from the case-control study to the full sample of emergency service users.

### Strong aspects of this design

The current study is one of very few studies in which data are systematically collected on both patient and service characteristics in a large metropolitan psychiatric emergency service. Getting to know different patterns and presentations of specific patient groups with a high risk of becoming emergency service users may result in developing early and more specific interventions to reduce the number of crisis situations and to improve adequate and timely care.

A further strength of our study is that it specifically examines factors leading coercive admissions. The care provided in Amsterdam Psychiatric Intensive Care Units is currently dominated by coercive treatment. This situation implies that patients with severe mental illnesses more often progress to stages in which acute, coercive treatment is warranted. It is clear that this is an undesirable trend, which not only leads to a reduction of patient autonomy but also has a negative effect on the prognosis of these disorders. Although the need for systematic longitudinal studies has frequently been advocated, only few studies have been able to follow-up this group of severely ill patients for longer periods of time. The fact that the Emergency Psychiatry Service (PESA) covers all psychiatric crisis consultations in Amsterdam, and contains the possibility of monitoring service use across the different mental health services enables such a design in Amsterdam.

## Competing interests

The authors declare that they have no competing interests.

## Authors' contributions

LvdP conceived and designed the study with contributions from NM, AB, RS and JD. LvdP, IV, and CB were responsible for the data collection. LvdP, RS, NM, LdH, AB and JD participated in the Steering Committee of the study and revised this manuscript critically for important intellectual content. RS, VK, LvdP transformed the Dutch study protocol into this manuscript. JD is head of the Research Department where the research is conducted and leads this research project. RS and JD are supervising all the ASAP studies. All authors provided comments, read and approved the final manuscript.

## Pre-publication history

The pre-publication history for this paper can be accessed here:



## Supplementary Material

Additional file 1 Grant approval 1. First of the two letters in which grant allocation is elucidated. Click here for file

Additional file 2 Grant approval 2. Second of the two letters in which grant allocation is elucidated. Click here for file

Additional file 3 Ethical approval. Letter from the medical ethical committee in which approval of the current study is confirmed. Click here for file
