# Supplementary material for: The Amsterdam Studies of Acute Psychiatry I (ASAP-I); A prospective cohort study of determinants and outcome of coercive versus voluntary treatment interventions in a metropolitan area
Source: BMC Psychiatry. 2008 May 14;8:35. doi: 10.1186/1471-244X-8-35 (PMC2413231; doi:10.1186/1471-244X-8-35)
Supplement: Additional file 1 — Grant approval 1. First of the two letters in which grant allocation is elucidated. [file 1471-244X-8-35-S1.pdf]

Stichting tot steun VCVGZ

Aan Metrum
t.a.v. dr. R.A. Schoevers
2^{e} Constantijn Huygensstraat 27
1054 AG Amsterdam

kenmerk : ST2137.Me
datum : 21 maart 2007
betreft : subsidieaanvraag

Geachte heer Schoevers,

In vervolg op de correspondentie in de afgelopen periode omtrent uw subsidieaanvraag "De betekenis van de dwangopname voor het behandelbeloop. Een onderzoek in de Spoedeisende Psychiatrie in Amsterdam" heeft het bestuur besloten uw aanvraag te honoreren..

Het toegekende subsidiebedrag, ad € 80.000, wordt in drie gedeelten uitbetaald, te weten 40% bij aanvang, 30% bij de eerste tussen rapportage en 30% bij de eindrapportage.

Graag vernemen wij op welk rekeningnummer, onder welke vermelding, wij de subsidie kunnen overmaken

Met vriendelijke groet,

drs. F.A.M. Meuwese
