# Supplementary material for: The Amsterdam Studies of Acute Psychiatry I (ASAP-I); A prospective cohort study of determinants and outcome of coercive versus voluntary treatment interventions in a metropolitan area
Source: BMC Psychiatry. 2008 May 14;8:35. doi: 10.1186/1471-244X-8-35 (PMC2413231; doi:10.1186/1471-244X-8-35)
Supplement: Additional file 2 — Grant approval 2. Second of the two letters in which grant allocation is elucidated. [file 1471-244X-8-35-S2.pdf]

Mentrum

Amsterdam, 17-05-2004

Geachte Professor Dekker,
Hoofd Onderzoek
Professor Klinische Psychologie VU
Betreft: De kans op een IBS opname in Amsterdam. Een onderzoek naar variabelen die de kans op een opname met Inbewaringstelling beinvloeden

Bij deze deel ik u mede dat voor uw afdeling voor het crisisdienst onderzoek 'De kans op een IBS opname in Amsterdam. Een onderzoek naar variabelen die de kans op een opname met Inbewaringstelling beinvloeden' vanuit Mentrum een bedrag van €250.000 euro in de komende jaren is gereserveerd.

Met hoogachting,

drs. Diana Monissen
Mentrum, Raad van Bestuur
