# Supplementary material for: The Amsterdam Studies of Acute Psychiatry I (ASAP-I); A prospective cohort study of determinants and outcome of coercive versus voluntary treatment interventions in a metropolitan area
Source: BMC Psychiatry. 2008 May 14;8:35. doi: 10.1186/1471-244X-8-35 (PMC2413231; doi:10.1186/1471-244X-8-35)
Supplement: Additional file 3 — Ethical approval. Letter from the medical ethical committee in which approval of the current study is confirmed. [file 1471-244X-8-35-S3.pdf]

Datum binnenkomst: 25/3/05

METiGG

Stichting Medisch-Ethische Toetsingscommissie Instellingen Geestelijke Gezondheidszorg

Onderzoeksnummer: 5206

Agendanummer : 28.8.2

GGZ Mentrum
drs. W. Mulder, Geneesheer Directeur
Postbus 75848
1070 AV AMSTERDAM

Kenmerk:	05.134/HB/ls
Datum :	25 maart 2005
Betreft:	De kans op een IBS opname in Amsterdam. Een onderzoek naar variabelen die de kans op een opname met inbewaringstelling beïnvloeden
Onderz. nr.:	5206
Indiening:	5 januari 2005

Geachte heer Mulder,

Op grond van artikel 2, tweede lid, onder a en artikel 16 van de *Wet medisch-wetenschappelijk onderzoek met mensen* (WMO), heeft onze commissie zich beraden over bovengenoemd onderzoeksprotocol.
De commissie heeft daarbij de in de bijlage genoemde stukken beoordeeld.

In deze studie worden determinanten onderzocht die van invloed zijn op het verloop van de behandeling van patiënten die zijn aangemeld bij de Amsterdamse crisisdienst. Het onderzoek bestaat uit drie tranches.
Deel I is de opzet van een registratie van (goeddeels bestaande) gegevens van een cohort van patiënten die zijn behandeld door de Amsterdamse crisisdienst in de loop van één jaar. Dit deel valt niet onder de WMO.
De onderstaande goedkeuring betreft de tweede tranche: interviews met twee groepen patiënten uit dit cohort (2 x 125 personen). De gegevens uit de crisisconsulten zullen indien mogelijk worden aangevuld met gegevens van GG & GD.

Tranche drie zal een follow-up op tranche twee zijn.

Behandeling van het onderzoek
De behandeling vond plaats in de vergaderingen van 18 januari, 15 februari en 15 maart jl. in de kamer Noord van de METiGG. De onderzoekers waren in de februarivergadering uitgenodigd voor overleg en het geven van een toelichting.
Er is met de onderzoekers gesproken over het beheer en koppeling van patiëntgegevens van de crisisdienst en de benadering van patiënten voor het afnemen van de vragenlijsten. Daarover is voldoende duidelijkheid verkregen.
Voor de koppeling met de gegevens GG & GD zal toestemming bij de registratiekamer

Kamer Noord
Postbus 86
3500 AB Utrecht
Da Costakade 45
T 030 - 295 94 34
F 030 - 297 11 11
E HansvanderBaan@metigg.nl

Kamer Zuid
Postbus 1078
5602 BB Eindhoven
Veldmaarschalk Montgomerylaan 333
T 040 - 232 97 61
F 040 - 244 00 86
E albertbollen@metigg.nl

K.v.K. nr. 30192997
Rabobank Eindhoven 16.24.47.884
Erkend door de CCMO

worden gevraagd. Als over deze koppeling nader is geregeld zal de werkwijze opnieuw aan onze commissie worden voorgelegd. Dit deel valt daarom vooralsnog buiten dit besluit.

Er met de onderzoekers ook gesproken over de noodzaak van alle vragenlijsten gelet op de eventuele belasting voor de deelnemers. De informatie voor de deelnemers is op enkele punten aangepast. Er zullen geen patiënten met een Rechterlijke Machtiging aan dit onderzoek deelnemen.

Patiënteninformatie
Gelet op het bepaalde in artikel 6, derde t/m zevende lid, van de WMO is de commissie van oordeel dat de proefpersonen op een adequate, volledige en begrijpelijke wijze over het onderzoek worden geïnformeerd. De goedkeuring betreft de versie die is meegestuurd met de brief van 11 maart 2005.

De verzekering
De commissie heeft vastgesteld dat op correcte wijze uitvoering is gegeven aan de verzekeringsplicht als neergelegd in artikel 7 van de WMO, zoals nader uitgewerkt in het Besluit van 23 juni 2003, houdende regels inzake de verplichte verzekering bij medisch-wetenschappelijk onderzoek met mensen (Besluit verplichte verzekering bij medisch-wetenschappelijk onderzoek met mensen).

Overweging en besluit
De commissie overweegt dat aan alle in artikel 3 van de WMO genoemde voorwaarden is voldaan. De commissie besluit positief over uitvoering van dit onderzoek in (zie bijlage).

Verplichtingen
De verrichter van het onderzoek dient rekening te houden met de volgende verplichtingen:
- Melding aan de commissie van de start en het einde van het onderzoek;
- Indiening van amendementen;
- Melding van ongewenste voorvallen (waaronder in ieder geval artikel 10 WMO);
- Melding voortijdige beëindiging onderzoek met de reden;
- Het verlies van de geldigheid van voorliggend oordeel indien de start van het onderzoek niet binnen 1 jaar plaats vindt;
- De melding van de resultaten onderzoek na afronding;
- Een verzoek om vergunning bij deelname van eventueel andere instellingen aan het onderzoek;
- Een jaarlijks voortgangsverslag.

Beroep
Ingevolge artikel 23 van de Wet medisch-wetenschappelijk onderzoek met mensen (WMO)

Kamer Noord
Postbus 86
3500 AB Utrecht
Da Costakade 45
T 030 - 295 94 34
F 030 - 297 11 11
E HansvanderBaan@metigg.nl

Kamer Zuid
Postbus 1078
5602 BB Eindhoven
Veldmaarschalk Montgomerylaan 333
T 040 - 232 97 61
F 040 - 244 00 86
E [illegible]

K.v.K. nr.30192997
Rabobank Eindhoven 16.24.47.884
[illegible]

kan degene wiens belang rechtstreeks bij het besluit is betrokken, daartegen binnen zes weken na de dag waarop dit besluit bekend is gemaakt, beroep instellen bij de CCMO. Een dergelijk beroepschrift dient geadresseerd te worden aan: CCMO, Postbus 16302, 2500 BH Den Haag.

Erop vertrouwend u met dit schrijven van dienst te zijn geweest,

Met vriendelijke groet,

Dr. P.A. de Groot, voorzitter

Namens deze:

drs. H. van der Baan
secretaris METiGG kamer Noord

Kamer Noord
Postbus 86
3500 AB Utrecht
Da Costakade 45
T 030 - 295 94 34
F 030 - 297 11 11
E HansvanderBaan@metigg.nl

Kamer Zuid
Postbus 1078
5602 BB Eindhoven
Veldmaarschalk Montgomerylaan 333
T 040 - 232 97 61
F 040 - 244 00 86
E albertbollen@metigg.nl

K.v.K. nr.30192997
Rabobank Eindhoven 16.24.47.884
Erkend door de CCMO

Onderzoek: De kans op een IBS opname in Amsterdam. Een onderzoek naar variabelen die de kans op een opname met inbewaringstelling beïnvloeden
Onderzoeksnummer: 5206

Besluit METiGG kamer Noord d.d. 25 maart 2005

1. Bij de beoordeling zijn de volgende documenten besproken:

- Aanbiedingsbrief d.d. 5-01-2005 met bijlagen:
  - aanbiedingsbrief d.d 5 januari 2005
  - Protocol IBS opname Amsterdam
  - bijlage a: Informed Consent Procedure
  - bijlage b: Meetinstrumenten
  - bijlage c: Verrekeningspolis
  - bijlage d: CV van hoofdonderzoeker
  - bijlage e: CV van onafhankelijk arts
  - bijlage f: CCMO formularia
  - bijlage g: samenvatting onderzoek
  - diskette + CCMO formularia plus abstract

- brief van 8 februari met toelichting en antwoorden op vragen met bijlagen :
  - aanvullingen op het onderzoeksprotocol (de punten 6.4 en 7.4 inzake gegevensbeheer en bescherming van privacy)
  - testprocedure interviews tranche twee
  - specificatie domeinen
  - oordeel Research Commissie van Mentrum

- brief van 11 maart 2005 met nadere toelichting op vragen en bevestiging afspraken uit vergadering van februari 2005:
  - bijgestelde patiënteninformatie

2. De samenstelling van de commissie ten tijde van de beoordeling is als volgt:

de heer dr. P.A. de Groot	Voorzitter
dr. J.W.B.M. van Berkestijn	Psychiater
drs. G. de Bruijn	Klinisch psycholoog/psychotherapeut
mw. dr. P.M.J. Haffmans	Farmacoloog
mr. W.H. Neisingh	Jurist
dr. F.W. Wilmink	Psychiater, methodoloog
drs. J. van den Berg	Verpleegkundige
drs. H. van der Baan	Medisch socioloog/ambtelijk secretaris
